# Tendon Reattachment to Bone in an Ovine Tendon Defect Model of Retraction Using Allogenic and Xenogenic Demineralised Bone Matrix Incorporated with Mesenchymal Stem Cells

**DOI:** 10.1371/journal.pone.0161473

**Published:** 2016-09-08

**Authors:** Tanujan Thangarajah, Shirin Shahbazi, Catherine J. Pendegrass, Simon Lambert, Susan Alexander, Gordon W. Blunn

**Affiliations:** 1 The John Scales Centre for Biomedical Engineering, Institute of Orthopaedics and Musculoskeletal Science, Division of Surgery and Interventional Science, University College London, The Royal National Orthopaedic Hospital Trust, Brockley Hill, Stanmore, Middlesex, HA7 4LP, United Kingdom; 2 The Shoulder and Elbow Service, The Royal National Orthopaedic Hospital, Stanmore, HA7 4LP, United Kingdom; National University of Ireland - Galway, IRELAND

## Abstract

**Background:**

Tendon-bone healing following rotator cuff repairs is mainly impaired by poor tissue quality. Demineralised bone matrix promotes healing of the tendon-bone interface but its role in the treatment of tendon tears with retraction has not been investigated. We hypothesized that cortical demineralised bone matrix used with minimally manipulated mesenchymal stem cells will result in improved function and restoration of the tendon-bone interface with no difference between xenogenic and allogenic scaffolds.

**Materials and Methods:**

In an ovine model, the patellar tendon was detached from the tibial tuberosity and a complete distal tendon transverse defect measuring 1 cm was created. Suture anchors were used to reattach the tendon and xenogenic demineralised bone matrix + minimally manipulated mesenchymal stem cells (n = 5), or allogenic demineralised bone matrix + minimally manipulated mesenchymal stem cells (n = 5) were used to bridge the defect. Graft incorporation into the tendon and its effect on regeneration of the enthesis was assessed using histomorphometry. Force plate analysis was used to assess functional recovery.

**Results:**

Compared to the xenograft, the allograft was associated with significantly higher functional weight bearing at 6 (P = 0.047), 9 (P = 0.028), and 12 weeks (P = 0.009). In the allogenic group this was accompanied by greater remodeling of the demineralised bone matrix into tendon-like tissue in the region of the defect (p = 0.015), and a more direct type of enthesis characterized by significantly more fibrocartilage (p = 0.039). No failures of tendon-bone healing were noted in either group.

**Conclusion:**

Demineralised bone matrix used with minimally manipulated mesenchymal stem cells promotes healing of the tendon-bone interface in an ovine model of acute tendon retraction, with superior mechanical and histological results associated with use of an allograft.

## Introduction

The muscles of the rotator cuff (subscapularis, supraspinatus, infraspinatus, and teres minor) play an important role in normal glenohumeral motion and stability. Some tears (deemed ‘irreparable rotator cuff tears’) cannot be repaired primarily to bone despite conventional techniques of mobilization and soft-tissue releases because of their size and retraction [1]. They are associated with atrophy and fatty infiltration of the associated rotator cuff muscles: this is reflected in an alteration of their pennation angles, which results in impaired muscle contraction and altered joint biomechanics [2, 3]. Left untreated, irreparable tears frequently lead to cuff tear arthropathy in susceptible individuals [4].

Poor biological healing following rotator cuff repair is a considerable problem, with failure of tendon-bone fixation occurring in up to 26% of small to medium tears and up to 94% in large and massive tears [5–9]. The cause of the high retear rate is multifactorial and may be attributed to the older age of the patient, quality of the tissue, chronicity and size of the tear, muscle atrophy, fatty infiltration, bone mineral density, and repair technique (single vs double row repair) [10–13]. In selected patients, mechanical and biological enhancement of the tendon-bone interface may be integral to a successful outcome following surgery [14]. Surgical options for treating irreparable rotator cuff tears include debridement +/- partial repair (arthroscopic or open), tendon transfer, and non-anatomic arthroplasty. The best patient outcomes are associated with tears that fully heal, however the results of surgery are varied and failure has been demonstrated to occur in up to 94% of non-arthroplasty cases [5, 15]. Current tissue engineering strategies to address this include scaffolds and biological factors, which can be used either in isolation or combination [16]. Demineralised bone matrix (DBM) is a collagen-based scaffold that is osteoinductive via endochondral ossification [17]. It is manufactured by removing the mineral component of bone tissue and has been shown to regenerate a functional enthesis with the formation of calcified and non-calcified cartilage interfacing bone and tendon with ‘Sharpey’s-like’ collagen fibres [18]. A sustained release of growth factors, including bone morphogenic proteins (BMPs), is thought to be responsible for this [17]. BMPs found in DBM have been shown to result in differentiation of mesenchymal stem cells (MSCs) into osteoblasts, chondrocytes and tenocytes but the direct effect of growth factors on intact tendon tissue is unknown [19–23].

The purpose of this study was to evaluate the effect of allogenic and xenogenic DBM used with autologous minimally manipulated MSCs (mmMSCs) on regeneration of the tendon-bone interface in an ovine model of acute tendon retraction. Since xenografts are cheaper and more readily available than their allogenic derivatives we wanted to assess their potential for augmenting tendon-bone healing [24]. We hypothesized that augmentation of a healing tendon-bone interface with DBM and mmMSCs would result in improved function, and restoration of the native enthesis, with no difference between xenogenic and allogenic scaffolds.

## Materials and Methods

### Study Design

All animal work was conducted in accordance with a Project License protocol accepted under the UK Home Office Animals (Scientific Procedures) Act 1986 and all efforts were made to minimize suffering. Ten skeletally mature non-pregnant female Friesland ewes, 2 to 3 years old, weighing between 78 and 97 kg were selected for this study. All 10 animals underwent surgical excision of the distal 1 cm of the right hind limb patellar tendon; the defect was repaired using a strip of xenogenic (porcine)/allogenic (ovine) DBM and mmMSCs. Animals were monitored continually throughout the experimental procedure by assessing baseline observations such as heart rate, blood pressure, and respiratory rate. All procedures were carried out by one surgeon. In keeping with the regulations set out by the UK Home Office Animals (Scientific Procedures) Act 1986, a control group was not used in this study since previous work has shown that a tendon defect does not heal spontaneously, rendering the animals lame [25, 26]. Animals were freely mobilized in individual pens with a constant supply of natural light and fed on hay. Supplementary minerals and salts were provided to maintain the remaining dietary requirements. At 12 weeks all animals were euthanised using intravenous Sodium Pentobarbital, following which specimens were retrieved. Force plate analysis, radiographs, pQCT scans, and histological analysis were performed.

### DBM manufacture

DBM derived from cortical bone was manufactured according to Urist’s protocol, with modifications [17]. Six tibiae from three skeletally mature female ewes (allograft) and sows (xenograft) were harvested immediately post euthanasia; all soft tissues and periosteum were stripped from the bone surface. Proximal and distal ends of each tibia were excised and the shafts were cut into three longitudinal sections corresponding to the three surfaces of the prismatic shape of the tibia using a diamond edged band saw (Exact, Hamburg, Germany). The resulting bone strips were then demineralized in 0.6 N hydrochloric acid (HCL) at room temperature. Demineralization was confirmed by taking radiographs (300 seconds, 30 kV, Faxitron Corporation, Illinois, USA). This was followed by prolonged washing in phosphate buffered saline (PBS) and lyophilisation (BOC Edwards, Crawley, West Sussex, UK) for storage. Strips measuring 3–4 mm in thickness, 20 mm (+/- 2 mm) wide, and 15 cm long were cut and sterilized by gamma irradiation at a dose of 25 KGrays (Isotron, Reading, UK). Samples were rehydrated at the time of surgery in normal saline for 45 minutes prior to use.

### Bone marrow aspiration

In order to deliver MSCs to the surgical site without expanding them using traditional time-consuming tissue culture techniques, we used the buffy layer from centrifuged autologous bone marrow aspirate obtained from the iliac crest at the time of surgery [27, 28]. Cells obtained in this manner are deemed ‘minimally manipulated MSCs’ (also known as bone marrow aspirate concentrates) and entail lower costs, faster processing, and less chance of an immunogenic reaction [29, 30]. Following the induction of anaesthesia, 20 ml of marrow was aspirated from the contralateral iliac crest in small fractions to reduce the degree of contamination by peripheral blood. Several aspirations were made through the same skin incision with each being spaced approximately 1 cm apart to avoid dilution from aspiration in the previous channel. All aspirates were stored in vials containing heparin and then concentrated by centrifugation with Ficoll for 30 minutes [27]. The buffy layer containing mmMSCs was returned to the operative room after 45 minutes.

### Surgical Procedure

**Fig 1:**
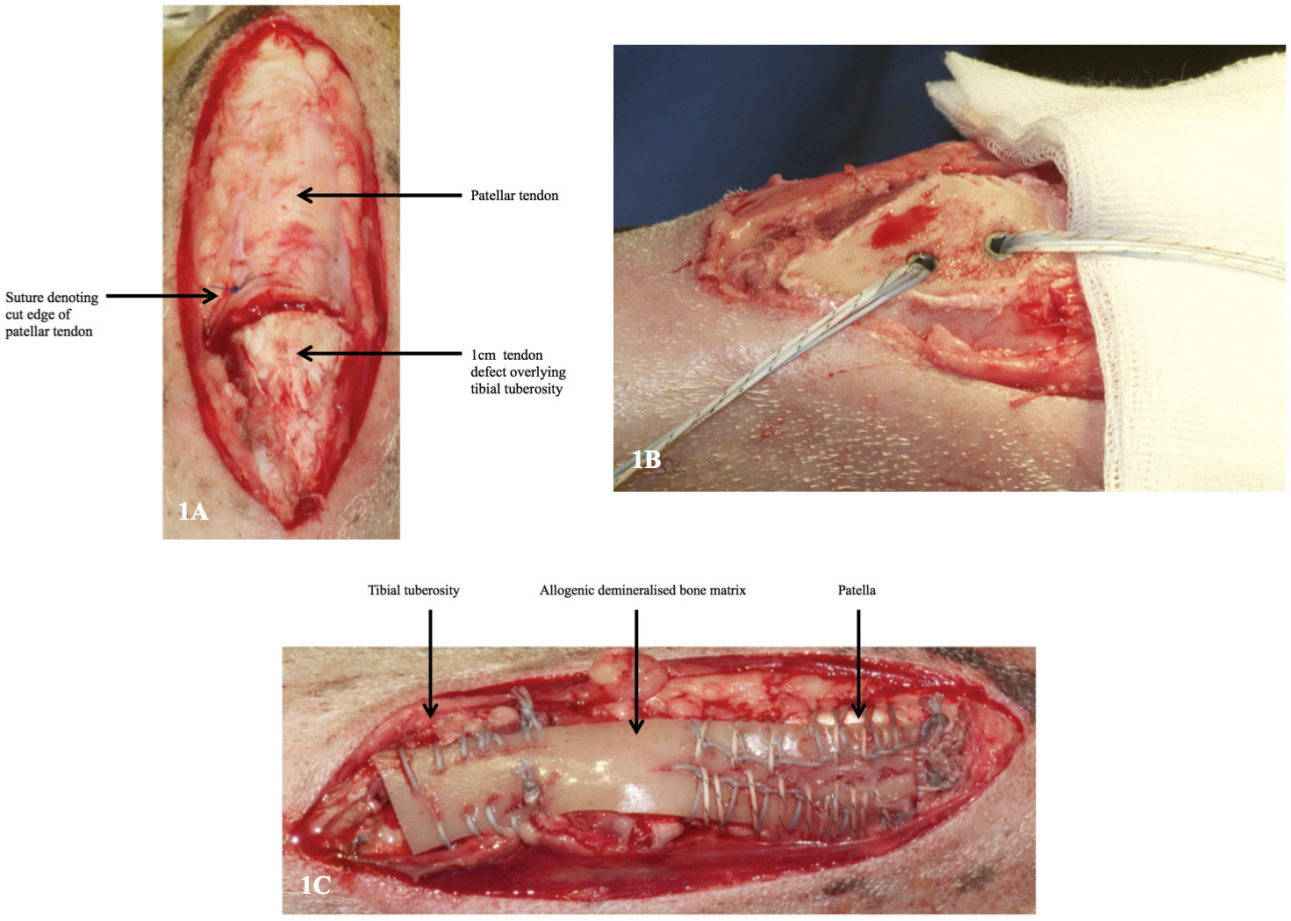
A. Ovine patellar-tendon defect. B. Osteotomised tibial tuberosity with two suture anchors *in situ*. The defect was repaired with allograft in five sheep and with xenograft in the remaining five sheep. An interlocking Krakow suture technique was used to secure the DBM strip to the remaining proximal patellar tendon using a #2 FiberWire (Fig 1C). The distal end of the DBM strip overlying the tibial tuberosity was secured to the prepared flat surface using the two anchors and its margins sutured to the surrounding tissue to ensure consistent and complete contact with the bony footprint. The dimensions of the DBM strip were adjusted to match the length and width of the host patellar tendon prior to repair: averaged 20 mm wide and 100 mm long. The surgical wound was closed in layers using absorbable Vicryl sutures. The sheep were allowed to move freely postoperatively without restraint. Animals received intravenous Buprenorphine at a dose of 0.006–0.01 mg/kg for a maximum of four days after surgery. Digital lateral X-ray radiographs of both hind limbs were taken at 12 weeks. Fig 1C. Ovine patellar tendon defect repaired with allogenic DBM.

An acute tendon retraction model was developed from a pre-existing ovine model used to examine tendon-bone healing [18]. All animals were sourced from The Royal Veterinary College (Hatfield, Hertfordshire, UK). Anaesthesia was induced with intravenous Midazolam (2.5 mg) and Ketamine Hydrochloride (2 mg/kg) and then maintained using 2% Isoflurane mixed with pure oxygen via an endotracheal tube. A longitudinal skin incision was made over the right stifle joint proximal to the upper pole of the patella to a point distal to the tibial tuberosity. The patellar tendon was identified and a transverse strip of the distal 1 cm was excised from its attachment (Fig 1A). Complete surgical avulsion of the patellar tendon insertion was confirmed by retraction of the proximal patellar tendon from the tibial tuberosity. The tendon insertion footprint was prepared by osteotomising the superficial 3 mm of bone with a sagittal saw parallel to the long axis of the tibia, and two 3.2 mm drill holes were made 1 cm apart on the prepared flat bone surface into which two 5.5 mm suture anchors (Corkscrew, Arthrex, Naples, Florida, USA) for reattachment of the DBM strip were inserted using a standard surgical technique (Fig 1B). The buffy layer was mixed with the fibrinogen component of a fibrin sealant (Tisseel, Baxter Health Care, Berkshire, United Kingdom) and then mixed with the thrombin component and applied onto the osteotomy site. Delivering MSCs in this way has been shown not to interfere with their proliferation or viability [31, 32].

### Force Plate Analysis

Animals underwent force plate analysis pre-operatively and at 6, 9, and 12 weeks postoperatively as this has been demonstrated to be an appropriate marker of functional recovery [18]. Twelve readings of each hind limb were taken by walking the animals over a force plate (Kistler Biomechanics Limited, Alton, UK) in a gait analysis laboratory. The mean peak vertical component of the ground reaction force (GRFz) of each hind limb was obtained and normalized for weight (Fmax/weight). Functional weight bearing (FWB) was expressed as the mean GRFz of the right hind limb as a percentage of the left (control) limb. Improvements in FWB were compared statistically between groups at each time point.

### Peripheral quantitative computed tomography

Peripheral quantitative computed tomography (pQCT) scanning was performed to examine for ossification within the patellar tendon, the DBM and surrounding tissues; 5 mm CT slices were taken through the tibial tuberosity, patellar tendon/DBM and patella. pQCT was undertaken, when specimens were retrieved at 12 weeks, using an XCT 2000 Bone Scanner (Stratec Medizintechnik Gmbh, Germany) with Software version 6.20.

### Histology

At 12 weeks the patella-patellar tendon/DBM graft-tibial tuberosity complex was harvested. Visual assessment of the tissue during the dissection, and pQCT were undertaken to determine if ossification had occurred at the margins of the graft. Samples were fixed in 10% formal saline and underwent ascending graded alcohol dehydration, defatting in chloroform, and embedding in LR White Hard Grade Resin (London Resin Company Limited, Reading, UK). Sections were cut, ground, and polished to 70–100 μm before staining with Toluidine Blue and Paragon.

**Fig 2:**
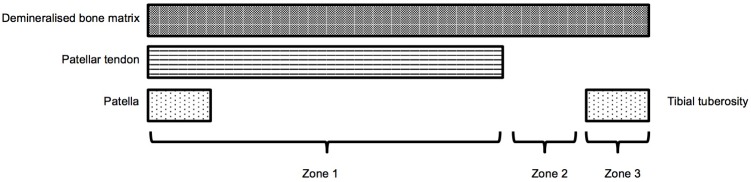
Three morphological zones examined histologically. Zone 1: DBM-patellar tendon interface; Zones 2: DBM in the region of the tendon defect; Zone 3: DBM neo-enthesis, examining the area of the new tendon enthesis over the tibial tuberosity.

Three distinct zones were defined and evaluated by two observers blinded to the origin of the graft (Fig 2). These comprised: zone 1, DBM-patellar tendon interface; zone 2, DBM in the region of the tendon defect; zone 3, DBM neo-enthesis, (the area of the new tendon enthesis over the tibial tuberosity). Sections were assessed for new bone formation, inflammatory cells, cellularity, vascularization and collagen fibre crimp.

Semi-quantitative analysis was undertaken for interactions between DBM and the patellar tendon (zone 1), remodeling of the DBM in the tendon defect (zone 2), and the formation of a neo-enthesis (zone 3). In Zone 1, four separate equally spaced regions were analysed in order to comprehensively examine the entire length of the DBM-patellar tendon interface. The maximum score for this area was therefore 20, in contrast to Zones 2 and 3 that could each yield a maximum score of 5. Tables 1 and 2 describe the scoring criteria used for these assessments.

**Table 1:** Criteria for semi-quantitative analysis of demineralised bone matrix remodelling (Zones 1 and 2).

Score	Criteria
1	DBM still present with no evidence of tendon integration
2	DBM: very little evidence of remodelling and integration Collagen: poorly aligned, poorly organised, not parallel
3	DBM: partial remodelling Collagen: partially aligned in a parallel manner, partially organised
4	DBM: almost completely remodelled Collagen: mostly aligned in parallel fashion
5	Completely remodelled DBM Normal tendon: - Parallel aligned collagen fibres - Elongated fibroblast nuclei

**Table 2:** Criteria for semi-quantitative analysis of the tendon-bone interface (Zone 3).

Score	Criteria
1	No fibrocartilage No mineralized fibrocartilage
2	Fibrocartilage present No mineralized fibrocartilage
3	Fibrocartilage present Mineralized fibrocartilage present Disorganized arrangement
4	Fibrocartilage present Mineralized fibrocartilage present Organized graduation between distinct regions but no tidemark
5	Fibrocartilage present Mineralized fibrocartilage present Organized graduation between distinct regions with tidemark

### Statistical Analysis

Numerical data were inputted into SPSS software package, version 23 (SPSS Inc, an IBM Company, Chicago, Illinois). Mann Whitney U tests were used to compare data between groups, whilst Wilcoxon Signed Rank tests were used to assess differences within each group over time. Results were considered significant at the p < 0.05 level.

## Results

All animals survived the duration of the study and none had post-operative infection or failure of the fixation, as noted on radiographs. At 12 weeks, the gait pattern had returned to normal in both groups.

### Force Plate Analysis

**Fig 3:**
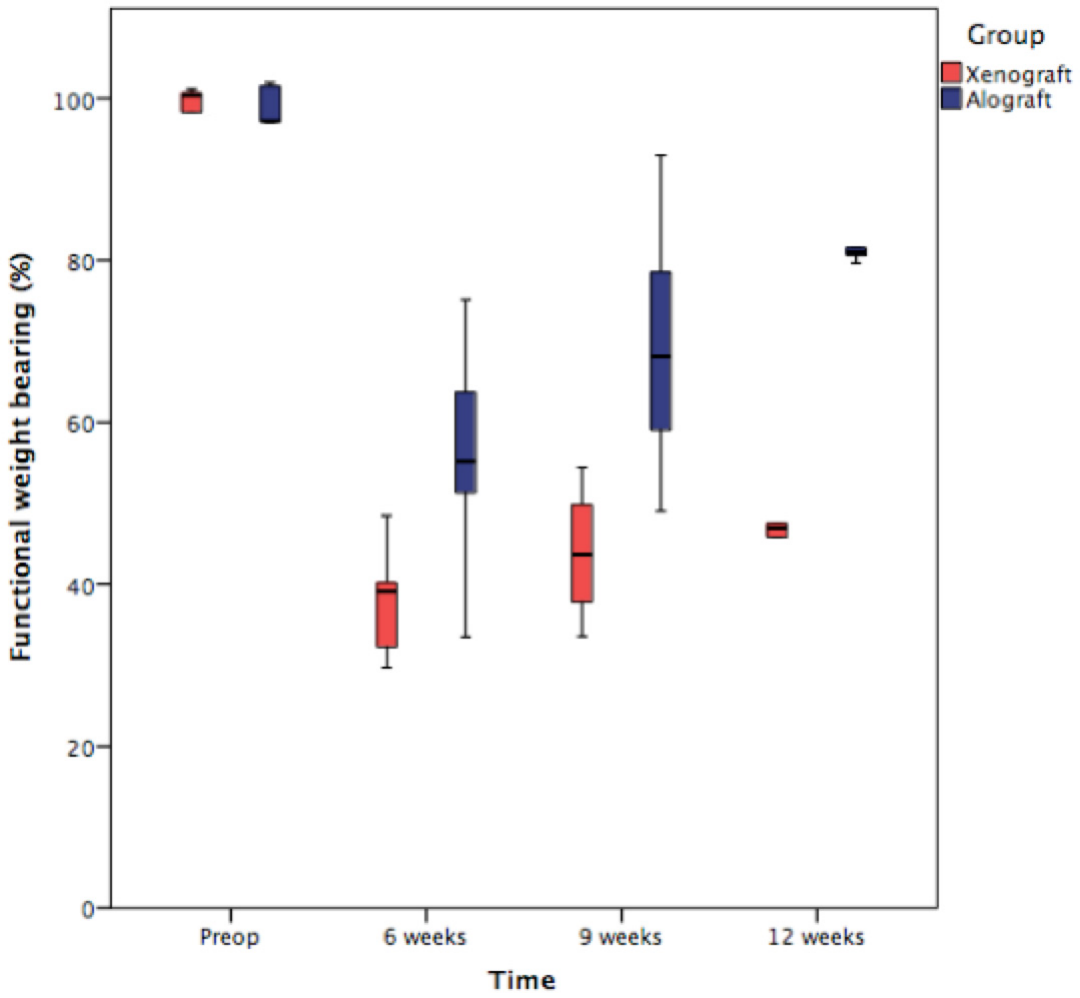
Box and whiskers plot showing percentage functional weight bearing of allogenic and xenogenic DBM groups at 6, 9 and 12 weeks.

In the allograft group, FWB reached a median of 55.2% (range, 33.5–75.1) at 6 weeks, 68.1% (range, 49.0–93.0) at 9 weeks, and 81.0% (range, 79.6–87.8) at 12 weeks. A significant improvement was noted between 6 and 9 weeks (p = 0.043). In the xenograft group, FWB reached a median of 39.1% (range, 29.7–48.5) at 6 weeks, 43.7% (range, 33.6–54.4) at 9 weeks, and 47.0% (range, 41.7–74.1) at 12 weeks. A significant improvement was noted between 6 and 9 weeks (p = 0.043). At 6, 9, and 12 weeks, FWB was significantly greater in the allograft group compared with the xenograft group (p = 0.047, 0.028, and 0.021, respectively) (Fig 3).

### pQCT Scans

None of the specimens showed any evidence of ossification within the DBM or the substance of the patellar tendon.

### Gross and Histological Findings

Normal post-operative scar tissue was present and there was no evidence of inflammatory reaction, infection or excessive granulation tissue. The DBM was well integrated into both the tibial tuberosity and patellar tendon and as no longer visible. The suture anchors remained well fixed with no evidence of pull out or migration. All suture materials were found intact with no evidence of rupture or failure.

**Fig 4:**
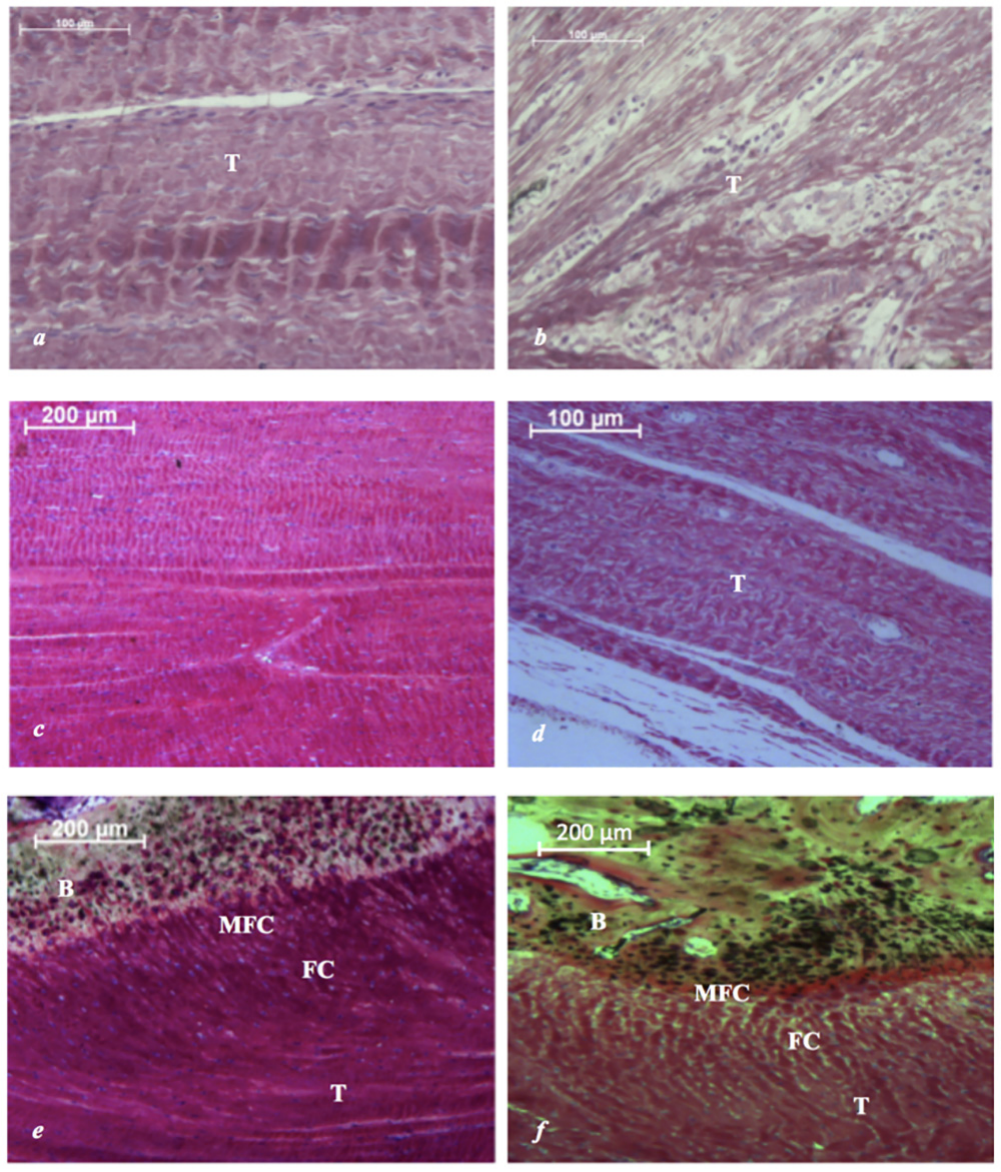
Photomicrographs showing appearance of DBM allograft and xenograft at 12 weeks. Specimens stained with Toluidine Blue and Paragon. (a) Zone 1: Patellar tendon-DBM allograft interface characterized by well-organized, crimped collagen fibres with elongated fibroblast nuclei. (b) Zone 1: Patellar tendon-DBM xenograft interface characterized by well-organized, crimped collagen fibres with elongated fibroblast nuclei. (c) Zone 2: Tendon defect with complete remodeling of DBM allograft and large areas of well-organized, crimped collagen fibres with elongated fibroblast nuclei. (d) Zone 2: Tendon defect with complete remodeling of DBM xenograft and large areas of well-organized, crimped collagen fibres with elongated fibroblast nuclei. (e) Zone 3: Allogenic DBM neo enthesis comprising tendon (T), fibrocartilage (FC), mineralized fibrocartilage (MFC) and bone (B). (f) Zone 3: Xenogenic DBM neo enthesis comprising disorganised tendon (T), fibrocartilage (FC), mineralized fibrocartilage (MFC) and bone (B).

At 12 weeks, both allogenic and xenogenic DBM was well integrated into the surrounding peritendinous tissue with evidence of neovascularization. In the allograft group, the DBM-patellar tendon interface (zone 1) and tendon defect (zone 2) appeared normal and both were characterized by well-organized, crimped collagen fibres with elongated fibroblast nuclei (Fig 4a and 4c). At the tendon-bone interface (zone 3), this was accompanied by mineralized fibrocartilage comprising chondrocytes surrounded by a mineralized matrix (Fig 4e). This direct type of enthesis, with a characteristic transition between bone, mineralized fibrocartilage, demineralized fibrocartilage and tendon was seen in both groups. However the xenograft DBM possessed fewer regions of mineralized fibrocartilage at the tendon-bone interface and displayed a more disorganised DBM-patellar tendon interface with fewer crimped collagen fibres (Fig 4b, 4d and 4f). No evidence of heterotopic ossification or inflammatory cells was observed in either group.

### Semi-quantitative Histology

At 12 weeks, the remodeling of the DBM was assessed using the criteria outlined in Table 1. In the region of the DBM-patellar tendon interface (zone 1), this scored a median of 14 (range, 11–16) in the allograft group and 12 (range, 11–15) in the xenograft group. This difference did not reach statistical significance (p = 0.147). At the tendon defect (zone 2), this scored a median of 4 (range, 3–5) in the allograft group and 3 (range, 2–4) in the xenograft group. In this region, the remodeled allograft resembled native tendon tissue significantly more than the xenograft (p = 0.015).

Analysis of the neo-enthesis (zone 3) was conducted using the criteria detailed in Table 2 with evaluation of the maturation of the enthesis and the presence of the four distinct zones (tendon, demineralized fibrocartilage, mineralized fibrocartilage and bone). This scored a median of 4 (range, 4–5) in the allograft group and 4 (range, 2–4) in the xenograft group. A significantly more mature neo-enthesis was formed in the allograft group (p = 0.039).

## Discussion

We have shown that allogenic and xenogenic DBM, used with mmMSCs, can regenerate an enthesis with favorable histological and functional properties in an ovine model of acute tendon retraction. All animals survived the duration of the study and there were no cases of post-operative infection or failure of the fixation. In both groups there was an improvement in functional weight bearing at all time points, but this was noted to be significantly greater in those animals that were treated with the allograft. At 12 weeks, the DBM had remodeled across the entire length of the patellar tendon including the defect. Histologically, it was characterized by well-organised crimped collagen fibres orientated in the direction of stress loading. The construct was well cellularised and vascularized throughout. At the tendon defect, the allograft was significantly more mature and had remodeled into tendon-like tissue with crimped collagen fibers orientated in the direction of the tendon to a greater extent than the xenograft. The tendon-bone interface had been restored in both groups and was defined by the four distinct regions that make up a direct enthesis. This was significantly more mature and organized in the allograft group. We speculate that the allogenic scaffold exhibited superior results compared to its xenogenic derivative because ovine bone has been demonstrated to contain a greater proportion of high-density bone particles than porcine bone, and a higher bone mineral content, bone mineral density, and fracture stress [33]. In this study, where a model of acute tendon retraction was used, it is plausible that the substantial forces borne by the scaffold may have resulted in repetitive micro-trauma of the weaker xenograft and consequently, inferior histological and functional outcomes.

Endochondral ossification has been proposed as a potential mechanism responsible for the development of the native enthesis [34]. It involves the initial release of growth factors followed by the secretion of a cartilaginous matrix that undergoes mineralization and remodeling to form bone [34]. This process is mediated by a number of growth factors including BMP-2 and BMP-7. These have been shown to specifically lead to bony ingrowth and osseous integration between tendon and bone, thereby strengthening the tendon attachment [35, 36]. DBM induces new bone formation by a similar process and may therefore represent a suitable scaffold to regenerate a functional tendon-bone interface [17]. Lovric et al [37] examined the effect of a DBM paste on healing of a bone tunnel during anterior cruciate ligament (ACL) reconstruction in a rodent model. At six weeks, peak load to failure was significantly higher in the DBM group when compared to non-augmented controls. Increased woven bone formation at the healing interface and greater expression of BMP-2 and BMP-7 were thought to be responsible for this. Histology did not show any evidence of fibrocartilage formation and the collagen present at the enthesis was disorganized and immature. Sundar et al [18] examined functional recovery of the ovine patella tendon-bone interface following tendon reattachment with a DBM scaffold. Repair failure was noted in 33% of non-augmented controls. In contrast, none of the repairs failed in the DBM group and the resultant enthesis comprised new bone and fibrocartilage. Earlier mobilization and superior function was noted at all time points.

Kilicoglu et al [38] assessed the effect of DBM on fixation of the extensor digitorum longus tendon within a proximal tibial bone tunnel in a rabbit model. At three weeks a greater proportion of Sharpey’s fibers, fibrocartilage and new bone was found in the DBM group when compared to non-augmented controls. To further evaluate the influence of DBM on tendon-bone healing within a bony tunnel, Hsu and Wang [39] used a rabbit model of ACL reconstruction. Similar to previous reports, DBM was associated with new bone formation and greater mineralized fibrocartilage.

MSCs have the potential to differentiate into tenocytes, chondrocytes, and osteoblasts, as well as being a rich source of growth factors that can enhance bone and tendon regeneration [40]. The delivery of MSCs to a specific anatomical site has traditionally been achieved by expansion *in vitro*, followed by either seeding onto a scaffold or direct implantation. Harvesting autologous bone marrow-derived MSCs is an appealing option since the aspirate can be concentrated and made available within 45 minutes, negating the need for time consuming culture-based expansion [27, 28, 40]. MSCs used in isolation to treat tendon defects have yielded variable results. Awad et al [41] examined the results of a full thickness patellar tendon defect repaired in rabbits using culture-expanded MSCs within a collagen gel. Compared to controls, the treatment group exhibited superior biomechanical properties but no differences in histological appearance. Juncosa-Melvin et al [42] evaluated the results of a collagen sponge, implanted with culture-expanded MSCs, that was used to repair a full thickness patellar tendon defect in rabbits. At 12 weeks, there was no difference in cellularity between the MSC group and non-augmented controls. No fibrocartilage was found in either group and the biomechanical properties of the MSC group were superior.

Our results of tendon-bone healing enhanced with DBM and mmMSCs contrast the aforementioned studies in that both the histological and functional properties were enhanced. We speculate that the increased amounts of BMPs present in DBM [37] caused the locally applied mmMSC population to differentiate down osteoblastic, chondrocytic, and tenocytic lineages [19–22]. The resultant interface resembled a direct enthesis containing organised fibrocartilage, and was able to resist the strong forces applied to it throughout the postoperative period without failure. Early postoperative weight bearing leading to mechanotransduction may have contributed to this since applying load to a developing enthesis causes bone at the insertion site to mature, increases fibrocartilage formation, and improves collagen organization [34]. Together, these factors contribute to the formation of a direct enthesis capable of resisting physiological forces.

We have used an ovine patellar tendon model to assess tendon-bone healing with DBM augmentation to simulate the clinical condition of the repair of an acutely retracted tendon. Despite this model not precisely representing chronic rotator cuff tears in a clinical setting due to the lack of degeneration associated with creating an acute tear, it does permit an accurate assessment of a healing enthesis as the extensor mechanism in a sheep has no supporting subsidiary structures and is entirely dependent upon the attachment of the patellar tendon to the tibial tuberosity. Detachment of supraspinatus in a sheep, and other animal models, would have had a substantially lesser effect on gait function as other components of the rotator cuff can compensate for its loss [43]. Other limitations of this study are associated with not quantifying the precise cell numbers in the mononuclear fraction, the influence of inter-species variation on the structure of DBM, and the lack of a control group. However, not treating the tendon defect would have rendered the animals lame, and therefore the study unethical, since previous work has shown that spontaneous healing does not occur [25, 26]. Future areas of study should examine the precise cellular mechanism involved in DBM enhancement of tendon-bone healing and compare it to other commercially available scaffolds, which are indicated for tendon repair back to bone following retraction.

## Conclusion

In conclusion, this is the first study to examine the effect of DBM used with mmMSCs on healing of a tendon-bone defect simulating acute tendon retraction. All animals were allowed unrestricted activity following surgery, which led to no failures. At 12 weeks, the tendon defect had successfully remodeled into tendon-like tissue and was accompanied by a direct enthesis. Functional weight bearing improved at all time points. Superior biomechanical and histological results were noted with the allogenic scaffold, highlighting the potential for DBM to be considered in the clinical setting to augment rotator cuff tendon-bone healing where retraction compounds conventional treatment strategies.

## Supporting Information

S1 Fig Supporting Information Raw Data. (XLSX)
